# Microglial Dysregulation and Suicidality: A Stress-Diathesis Perspective

**DOI:** 10.3389/fpsyt.2020.00781

**Published:** 2020-08-11

**Authors:** Paria Baharikhoob, Nathan J. Kolla

**Affiliations:** ^1^ Institute of Medical Science, University of Toronto, Toronto, ON, Canada; ^2^ Centre for Addiction and Mental Health (CAMH) Research Imaging Centre and Campbell Family Mental Health Research Institute, Toronto, ON, Canada; ^3^ Violence Prevention Neurobiological Research Unit, CAMH, Toronto, ON, Canada; ^4^ Department of Psychiatry, University of Toronto, Toronto, ON, Canada; ^5^ Waypoint Centre for Mental Health Care, Waypoint Research Institute, Penetanguishene, ON, Canada

**Keywords:** suicide, stress-diathesis model, microglia, neuroinflammation, post-mortem, positron emission tomography

## Abstract

According to the stress-diathesis model of suicidal behavior, completed suicide depends on the interaction between psychosocial stressors and a trait-like susceptibility. While there are likely multiple biological processes at play in suicidal behavior, recent findings point to over-activation of microglia, the resident macrophages of the central nervous system, as implicated in stress-induced suicidal behavior. However, it remains unclear how microglial dysregulation can be integrated into a clinical model of suicidal behavior. Therefore, this narrative review aims to (1) examine the findings from human post-mortem and neuroimaging studies that report a relationship between microglial activation and suicidal behavior, and (2) update the clinical model of suicidal behavior to integrate the role of microglia. A systematic search of SCOPUS, PubMed, PsycINFO, and Embase databases revealed evidence of morphological alterations in microglia and increased translocator protein density in the brains of individuals with suicidality, pointing to a positive relationship between microglial dysregulation and suicidal behavior. The studies also suggested several pathological mechanisms leading to suicidal behavior that may involve microglial dysregulation, namely (1) enhanced metabolism of tryptophan to quinolinic acid through the kynurenine pathway and associated serotonin depletion; (2) increased quinolinic acid leading to excessive N-methyl-D-aspartate-signaling, resulting in potential disruption of the blood brain barrier; (3) increased quinolinic acid resulting in higher neurotoxicity, and; (4) elevated interleukin 6 contributing to loss of inhibition of glutamatergic neurons, causing heightened glutamate release and excitotoxicity. Based on these pathways, we reconceptualized the stress-diathesis theory of suicidal behavior to incorporate the role of microglial activity.

## Introduction

Understanding the complex, multifactorial phenomena of suicidal behavior (SB) has long been a research priority. In 1999, Mann and colleagues were the first to introduce a stress-diathesis approach to suicide risk, which they denoted as the clinical model of SB. This model postulates that state-dependent “stress” in the form of psychosocial stressors and psychiatric illness(es) in combination with a trait-like “diathesis” to suicidal tendencies could result in SB (1). The stress component refers to the proximal risk factors that affect the timing and probability of suicidal acts, while the diathesis component describes distal risk factors influencing SB and may be considered independent of a psychiatric illness (2). This model is highly influential and has formed the framework for much of the contemporary research in suicidology; however, the model is not without limitations. Most importantly, it is difficult to discern the neurobiological or molecular underpinnings that influence the stress component and its interactions with the diathesis component.

The clinical model of SB has since been modified by the fields of neurobiology and genetics in order to incorporate a neurobiological basis. For example, studies have demonstrated that early life stressors or trauma, such as physical or sexual abuse, decrease serotonergic neurotransmission (3–6), which is associated with increased impulsivity and aggressive behavior in adults (7). Epigenetic regulation of genes involved with hypothalamic pituitary adrenal (HPA) axis responsiveness have also been implicated in the model, which results in a blunted cortisol response and reduced resilience in managing stress (8–15). These changes likely contribute to emotion dysregulation, high anxiety, impulsive/aggressive behavior, as well as impaired problem-solving and decision-making. Furthermore, early life stressors are also found to be associated with reduced cerebrospinal fluid (CSF) oxytocin levels (16). Neuropeptides, such as opioids, oxytocin, and vasopressin, play an important role in emotion regulation and are disrupted in individuals with borderline personality disorder, who are highly prone to engaging in SB (17).

Though the aforementioned findings regarding the neurobiological and genetic dimensions of suicide risk have undoubtedly been useful in improving the clinical model of SB, the most updated stress-diathesis orientated model that integrates and illustrates these findings (18) is by no means exhaustive. This is because there have been other molecular abnormalities described in relation to the risk of suicide that have not yet been incorporated in the model. In particular, several studies have recently suggested a relationship between microglial activation and SB. Specifically, the over-activation of microglia, which are the resident immune cells of the central nervous system (CNS), is posited to contribute to SB. In addition to traumatic and immune stimuli, microglia can be activated as a result of psychosocial stress (19–21). Chronic microglial activation may, therefore, also influence the interaction between the stress and diathesis components of the stress-diathesis model of SB, including contributing to an increased vulnerability to suicidal tendencies. The role of microglial dysregulation has not been discussed within the context of a stress-diathesis model. In this paper, we review the results from the original immunohistochemistry and neuroimaging studies that discuss microglial activity in relation to SB. We then incorporate the role of microglial activity in an updated stress-diathesis theory of SB.

## Phenotype and Function of Microglia

Microglia are the resident tissue macrophages of the CNS, representing 5 to 20% of the total glial cell population (22). In a healthy brain, “resting” or “quiescent” microglia have a small cell body with fine, ramified, and highly dynamic processes. This increased surface area allows microglia to continually scan their local environment for signs of potential threats. Upon recognition of harmful stimuli or infectious agents within the micro-environment, microglia rapidly become activated. This leads to a retraction of microglia protrusions and hypertrophy of their soma, giving the microglia an amoeboid-like shape (20). When activated, microglia release different inflammatory mediators depending on the state of activation.

Activated microglia can be divided into two states, the pro-inflammatory (M1) and anti-inflammatory (M2) pathways. The M1 pathway is triggered after neuronal injury has occurred and results in the release of a number of pro-inflammatory cytokines by microglia, including interleukin (IL)-1β, IL-6, tumor necrosis factor (TNF)-α, and nitric oxide (NO) (23). This is followed by the secondary response of the M2 pathway, which leads to the release of anti-inflammatory signals by microglia, namely IL-10, insulin-like growth factor 1, transforming growth factor beta, as well as various neurotrophic factors. The anti-inflammatory response of the M2 pathway is meant to restore homeostasis through the clearing of debris, reconstruction of the extracellular matrix, tissue repair, and angiogenesis. In a healthy immune system, both the M1 and M2 pathways are necessary, and the balance between the two states is part of a functional immune response (19, 23).

The nomenclature of the M1 and M2 pathways has, nevertheless, been challenged. This is because the states were defined based on the exposure of macrophages to stimuli *in vitro* and not *in vivo*. Thus, several scholars argue that the differentiation of the two states for activated microglia may be too reductionistic and instead hypothesize that microglial activation exists along a continuum (20, 24). With this point in mind, we will refer to microglial activation in general terms rather than specifying the M1 or M2 pathways for the purposes of this review.

## Psychosocial Stress, Microglia and Depressive-Like Behavior in Animal Models

According to several studies involving animal models, prolonged psychosocial stress can activate microglia (19). In fact, as evident in **Table 1**, at least 16 animal studies have shown that in the prefrontal cortex, exposure to different psychosocial stressors gives rise to increases in ionized calcium-binding adaptor molecule 1 (Iba-1) immunoreactivity, a specific marker for the morphological changes and expansion of microglia. These psychosocial stressors involve repeated social defeat (SD), varying unpredictable stress, prenatal stress, social isolation, and chronic restraint (25–37). Similar effects from psychosocial stressors were also evident in other areas of the brain, including the amygdala (25, 26), hippocampus (25–28, 30, 36, 38, 39, 41–47), nucleus accumbens (36, 37) and paraventricular nucleus (25, 26). The SD paradigm, which is described below, appears to have the most marked effect on Iba-1 immunoreactivity.

**Table 1:** Effect of psychosocial stress on microglial activity in prefrontal cortex.

Reference	Stress Type	Species	Effect on Iba-1^1^
(25–28)	Chronic social defeat, chronic unpredictable stress	Mice, rats	↑↑↑
(29, 30)	Chronic unpredictable stress, prenatal stress	Mice, rats	↑↑
(31–37)	Chronic unpredictable stress, chronic restraint, social isolation	Mice, rats	↑
(32, 38–40)	Chronic social defeat, chronic unpredictable stress, chronic restraint	Mice, rats	↔

^1^Microglial activity as measured by Iba-1 immunoreactivity. A ↑↑↑ effect represents a >70% increase in Iba-1 activity, a ↑↑ effect represents a 30-70% increase, a ↑ effect represents a 5-30% increase, and a ↔ represents no significant differences.

SD is considered an animal model of depression as it induces depressive-like behavior in animals following prolonged psychosocial stress. The paradigm is typically initiated when a male rodent is presented to the domicile of an older, more dominant and aggressive male. The less dominant intruder rodent is rapidly attacked and forced into submission, consistently exhibiting signs of distress. These signs involve elevated glucocorticoid activity, hyperthermia, and tachycardia (48). Chronic psychosocial stress induced by repeated exposure to this situation has also been shown to impose long-term, depressive-like behavioral changes in the intruder, including sensitivity to other stressors and signs of social avoidance, anhedonia, locomotor hypomotility, and metabolic changes (49–51).

Unlike in other animal models of depression, chronic, but not acute, administration of anti-inflammatory medications to rodents can be effective in reducing depressive-like manifestations. Four-week treatment with minocycline, a tetracycline antibiotic which inhibits the increase and activation of microglia, successfully reduces the depressive-like behavior of rats exposed to chronic stress. Minocycline normalizes Iba-1 immunoreactivity in the hippocampus and reduces the increase of mRNA levels of pro-inflammatory cytokines, such as IL-1β, IL-18, and IL-6 (34, 52). These results suggest that microglial activation may play a crucial role in the regulation of stress-induced depressive-like behavior.

These findings are supported by a number of other animal studies, which have shown that stress-induced depressive-like outcomes by SD may, in part, be mediated by microglial activation (40, 47, 53–56). Stein and colleagues, who reviewed these studies in depth, posit that chronic stress contributes to the over-activated states of microglia. The over-activation of microglia, as a result, leads to highly increased levels of pro-inflammatory mediators, cytotoxins, and reactive oxygen species (ROS), which may produce excessively pruned synaptic connections, causing cellular dystrophy and loss or reduced functioning of neuronal activity. The cellular damage as well as impaired neuronal activity may subsequently induce the development of depressive-like outcomes (57).

Depressive-like behavior, which is part of the stress component of the clinical model of SB, can be emulated by animal models, such as the aforementioned SD paradigm; however, the outcome, SB, *cannot* be reproduced by animals as there are no convincing animal models of SB. This is partially due to the notion that animals cannot arguably execute SB due to their inability to possess a will to die, to perceive the ultimate consequences of their suicidal acts, or to consider the emotional reactions of those who would survive their conceivable death (58, 59). Therefore, for this review, we turn our attention to microglial dysregulation in relation to suicidality in human studies.

Interestingly, in human post-mortem studies, microglial over-activation also appears to be implicated in the pathophysiology of SB. According to Steiner and colleagues, suicide victims with a prior history of either major depressive disorder (MDD) or paranoid schizophrenia (SCZ) have elevated levels of microglial activity in the dorsolateral prefrontal cortex (DLPFC), anterior cingulate cortex (ACC) and mediodorsal thalamus (MDT) (60). Moreover, in a study that measured Iba-1 immunoreactivity similar to the animal models above, the results demonstrated a significantly higher degree of microglial priming in the dorsal ACC white matter of depressed suicide victims (61). Other studies have theorized, likewise to Stein and investigators (57), that microglial dysregulation results in elevated levels of pro-inflammatory cytokines, such as IL-1β, IL-6, TNF-α, cytotoxins, and NOX2-derived ROS. These studies also propose that such mechanisms contribute to neurotoxicity, excitotoxicity, and heightened glutamate release, alterations of which may then lead to depressive-like behavior apposite to suicidality (62–64). The aforementioned literature, alongside other human studies evaluating microglial dysregulation in SB, will be described in detail later in this review.

## Methods

### Information Sources and Search Strategy

Potentially relevant studies were identified using the four electronic databases, SCOPUS, PubMed, PsycINFO, and Embase. Studies eligible for review included those concerning microglial activation and suicidal and self-harming acts in humans. They included human post-mortem and neuroimaging studies. Searches were performed between January 2006 and December 2019. We chose 2006 as the lower limit because it marked the year that the first paper addressing a possible relationship between microglial dysregulation and suicidality in humans was published (65). The following keywords were used for searches for each database: “microglia”, “microglial activation”, “microglial dysregulation”, “suicidality”, “suicidal behavior”, “suicide”, “suicide attempt”, “suicidal ideation”, “parasuicidal behavior”, “self-harming behavior”, and “self-harm”.

### Eligibility Criteria

Considering the different databases used for identifying studies, the abstracts and titles identified from the database searches were initially screened for eligibility using the following inclusion criteria: (1) original articles published between January 1, 2006, and December 31, 2019. Following this step, 44 were excluded from an initial 277 studies. This led to a remaining 233 studies, which were then screened using the following inclusion criteria: (1) studies that involved human subjects, (2) studies that were in English, and (3) studies that were not review articles. 121 studies were eliminated after this stage of screening, resulting in 112 articles.

The remaining 112 articles that met the aforementioned eligibility criteria were extracted to the reference management software, Zotero (Version 5.0.79). The full text of these articles was reviewed and included if they reported microglial dysregulation in the context of SB. All duplicate articles were subsequently deleted. This step in the screening process eliminated 103 articles, constituting a total of 9 studies that were included in this review.

### Study Selection

**Figure 1:**
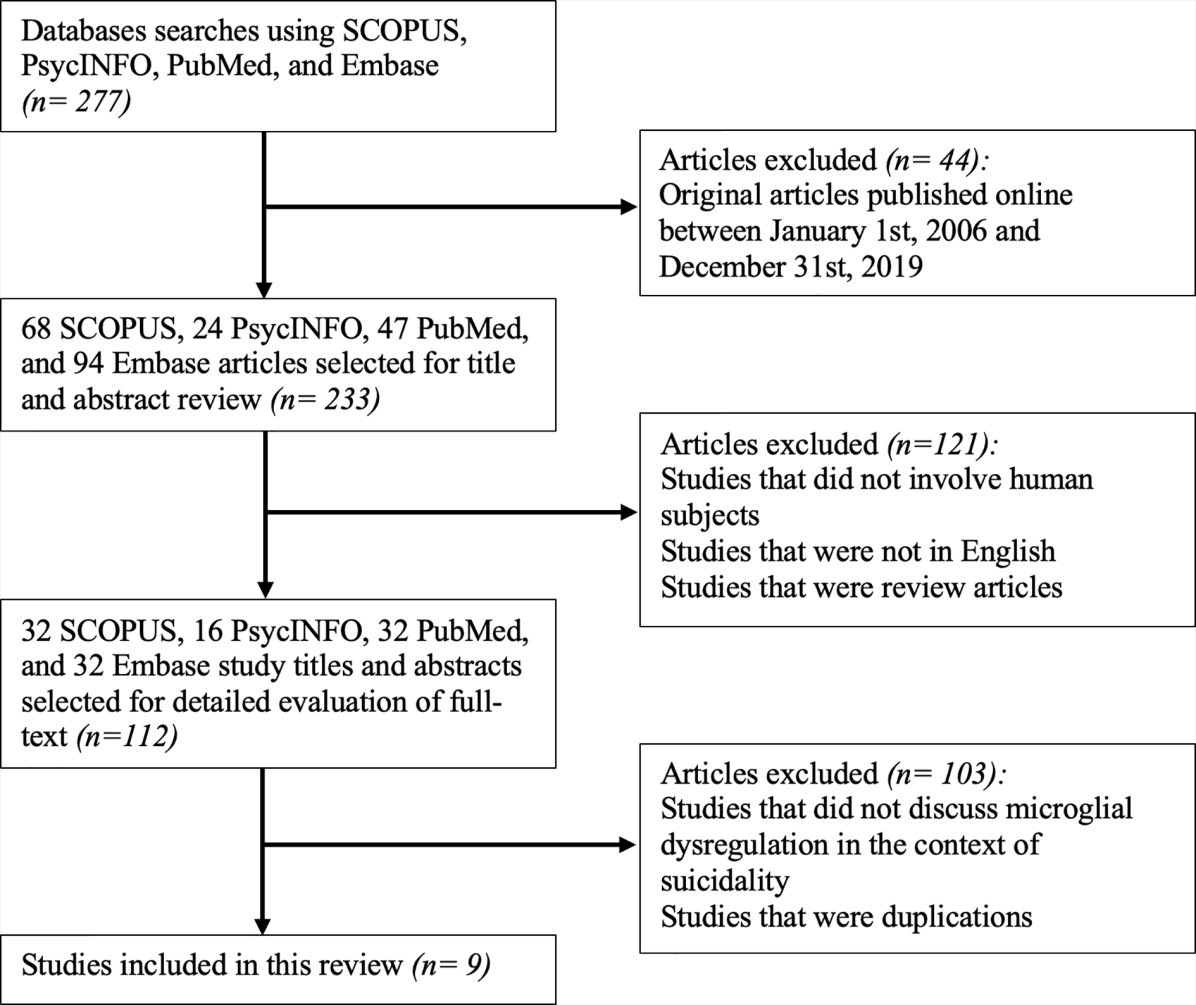
Flowchart for the inclusion of appropriate original articles. This flowchart showcases the selection process for the inclusion of articles for review according to the PRISMA statement (66).

The lead author was responsible for reviewing titles and abstracts following the database searches. The full text of articles that passed the first set of eligibility criteria were also reviewed by the lead author. If the relevance of an article was deemed questionable by the lead author, then the second author was responsible for reviewing the full text of the article and concluding its appropriateness for inclusion. The study selection process is summarized in **Figure 1**.

## Results

### Post-Mortem or Immunohistochemistry Studies Implicating Microglia and SB

Performing immunohistochemical analyses from post-mortem brain samples is an important technique for furthering our understanding of the neurochemical dimensions of complex psychiatric phenomena, including SB (67). Eight separate post-mortem studies utilized this technique to investigate potentially dysregulated microglial activity in SB, which can be observed in **Table 2**. Findings from four studies pointed to significant microglial activity in the brains of suicide victims compared with healthy control (HC) subjects (60–62, 65); two studies observed no significant differences in activated microglia between suicide victims and HC subjects or found a minor percentage of microglia changes (63, 64); one study observed decreased microglial activity in their non-suicide group (69); and, a final study found decreased microglial activity in suicide victims compared to HC subjects (68).

**Table 2:** Post-mortem studies of microglial activity in SB.

Reference	Brain bank	Study Population	Death from Suicide	Mean Age (years)	Immunoreactivity marker(s) for microglial activity	Brain region	Findings
(65)	MBB	9 SCZ (paranoid) 7 SCZ (residual) 16 HC	2 SCZ (paranoid)	SCZ suicide: 50 SCZ non-suicide: 51 HC: 58	HLA-DR	DLPFC, ACC, MDT, and hippocampus	Highly elevated microglial activity in ACC and MDT of suicide victims.
(60)	MBB	9 MDD 5 BD 12 SCZ (paranoid) 4 SCZ (residual) 10 HC	7 MDD 6 SCZ (paranoid)	SCZ suicide: 50 SCZ non-suicide: 55 AD suicide: 41 AD non-suicide: 51 HC: 54	HLA-DR	DLPFC, ACC, MDT, and hippocampus	Significant microglial activity observed in DLPFC (p=0.004), ACC (p=0.012), and MDT (p=0.004) of suicide victims.
(62)	MBB	7 MDD 5 BD 10 HC	7 MDD 5 BD	Suicide: 51 HC: 56	QUIN	sACC, aMCC, pACC	Significant microglial activity evident in sACC (p=0.003) and aMCC (p=0.015) of suicide victims with MDD.
(61)	DBCBB	20 UD 4 D-NOS	20 UD 4 D-NOS	Suicide: 46 HC: 39	IBA-IR, CD45	dACC white matter	Significantly increased microglial priming in dACC white matter of depressed suicide victims (p=0.030).
(63)	NYSPIBC	10 SCZ 8 AD 18 No Illness	3 SCZ 5 AD 3 No Illness	Suicide: 56 Non-suicide: 55	IBA-1, CD68	VPFWM, DPFWM	No difference in microglial activity between suicide victims and non-suicide victims in VPFWM or DPFWM.
(68)	MBB	6 UD 6 BD 10 HC	6 UD 6 BD	Suicide: 49 HC: 56	QUIN	Hippocampus subregions (CA1 and CA2/3)	Significantly decreased microglial activity in right CA1 subregion of both UD (p=0.048) and BD (p= 0.031) suicide victims.
(69)	MBB	15 MDD 12 BD 9 SCZ (paranoid) 9 SCZ (residual) 22 HC	9 MDD 7 BD 7 SCZ (paranoid) 1 SCZ (residual)	Suicide: 48 Non-suicide: 54 HC: 52	HLA-DR	DRN	Significantly decreased microglial activity in DRN depressed in non-suicide group compared to suicide victims with MDD or BD and controls (p=0.039).
(64)	DCEM, UF	26 AS 6 NSA 10 HC	26 AS	Not available	NOX2/MAC387	Cortex	Minor percentage of NOX2/MAC387 co-staining in cortex of AS victims.

AD, affective disorder; aMCC, anterior midcingulate cortex; AS, asphyctic suicide; BD, bipolar disorder or bipolar depression; CD45, cluster of differentiation 45; CD68, cluster of differentiation 68; dACC, dorsal anterior cingulate cortex; DBCBB, Douglas-Bell Canada Brain Bank; DCEM, Department of Clinical and Experimental Medicine; DLPFC, dorsolateral prefrontal cortex; D-NOS, depression not otherwise specified; DPFWM, dorsal prefrontal white matter; DRN, dorsal raphe nucleus; HC, healthy control; HLA-DR, human leukocyte antigen– DR isotype; IBA-IR, ionized calcium-binding adaptor molecule 1-immunoreactive; IBA-1, ionized calcium-binding adaptor molecule 1; MBB, Magdeburg Brain Bank; MDT, mediodorsal thalamus; MDD, major depressive disorder; NOX2/MAC387, NADPH oxidase and macrophage marker antibody; NYSPIBC, New York State Psychiatric Institute Brain Collection; pACC, pregenual anterior cingulate cortex; QUIN, quinolinic acid; sACC, subgenual anterior cingulate cortex; SCZ, schizophrenia; UD, unipolar disorder; UF, University of Foggia; VPFWM, ventral prefrontal white matter.

#### Positive Studies

The results from four studies point to a positive association between increased microglial activity and SB. To begin, in 2006, Steiner et al. published the first study with an incidental observation of increased microglial densities in the ACC and MDT of 2 paranoid SCZ suicide victims (65). A similar group followed up on this finding and went on to provide the first significant evidence, using HLA-DR staining, of higher microglial density in the DLPFC, ACC, and MDT of suicide victims who had MDD or paranoid SCZ (60). This positive relationship was similarly replicated in a later study using the endogenous modulator, quinolinic acid (QUIN), as an immunoreactivity signature. The authors from this study observed significantly increased density of QUIN-positive cells in the subgenual ACC and anterior midcingulate cortex of suicide victims with MDD (62). Furthermore, another paper observed increased microglial priming in the dorsal ACC white matter of depressed suicide victims through the use of Iba-1–immunoreactive and Cluster of Differentiation (CD45) staining (61). Altogether, these results suggest that increased microglial activity may be correlated with death by suicide and that mechanisms relating to increased QUIN production by activated microglia may underlie this association.

#### Mixed Studies

Two studies presented unclear conclusions that neither support nor refute the *a priori* hypothesis of a positive relationship between the degree of microglial over-activation and SB. A team examining Iba-1 with CD68 discovered no significant differences in the ventral prefrontal white matter or dorsal prefrontal white matter between suicide victims and non-suicidal victims after adjusting for multiple statistical tests. There was an effect of suicide on the density of activated phagocytes in the ventral prefrontal white matter compared with the dorsal prefrontal white matter. Nevertheless, considering that activated phagocytes in their study comprised both activated microglia and macrophages, making any conclusions about the exclusivity of microglial activity is difficult (63). Comparatively, a study utilizing NADPH oxidase and macrophage marker antibody (NOX2/MAC387) co-staining found a small percentage of this staining in the cortex of victims of asphyxia suicide. It remains unclear, however, whether this percentage was significantly different from the percentage of those who died by non-suicidal asphyxia or in HC subjects (64). These results preclude firm conclusions; however, the authors speculated that disruption of the blood brain barrier (BBB) due to higher QUIN levels as well as increased IL-6 production could be mechanisms associated with SB.

Finally, two studies found evidence of unchanged or reduced microglial activity in suicide cases compared to HC subjects. A paper from 2017 identified significantly decreased microglial activity, using HLA-DR staining, in the dorsal raphe nucleus of the non-suicidal depressed group in comparison to suicide victims with either MDD or bipolar disorder (BD), as well as HC subjects (69). Similarly, a study by Busse and colleagues revealed decreased microglial QUIN-immunoreactivity after *post hoc* tests in the right hippocampal CA1 field of suicidal patients with MDD or BD. These subjects, nevertheless, had an unchanged hippocampal volume size (68). The findings of these studies suggest that local anti-inflammatory and/or neuroprotective responses in these subjects may have influenced the extent of the microglial activation.

In summary, four of the eight post-mortem studies described above provided evidence of morphological alterations, predominantly increased cell density, in the microglia of the brains of suicide victims. These results support the theory of a positive relationship between microglial dysregulation and SB regardless of psychiatric diagnosis, implying that microglial-induced SB may be independent of psychiatric illness(es). However, the remaining four post-mortem studies had inconclusive findings, for example, unchanged or reduced microglial activity in subjects with SB. With this information in mind, more extensive post-mortem or immunohistochemistry investigations are required to make any definitive statements regarding neuroinflammation, specifically activated microglia, in suicide victims.

#### Limitations

There are some limitations to the post-mortem studies reviewed that must be mentioned. First, the microglial markers HLA-DR, Iba-1, and NOX2/MAC387 were limiting in that there was no suggestion of a molecular mechanism for which increased microglial activity may be related to SB. Moreover, with regards to the microglial marker, QUIN, the presence of released or secreted QUIN in the extracellular space and/or of protein expression or activity of the kynurenine (KYN) pathway enzyme QUIN phosphoribosyltransferease may have also influenced glutamatergic neurotransmission. These other sources of QUIN thereby eliminate our ability to conclude that strictly microglial QUIN influences neuromodulation and ultimately behavioral manifestations. Second, all of the studies had small sample sizes, especially with consideration to the number of suicide victims. Third, for many of the studies, brain samples were obtained from different brain banks and comprised patients who had differing lengths of psychiatric illnesses, causes of death, exposure to psychopharmacological interventions, and potentially other age-related brain pathologies.

### Positron Emission Tomography Study Discussing Microglial Activation in Relation to SB

The imaging modality positron emission tomography (PET) with the radioligand, [^11^C]-(R)-PK11195, is another useful tool for visualizing microglial activity in the CNS in various psychiatric disorders. This is because [^11^C]-(R)-PK11195 binds selectively to the translocator protein (TSPO) 18 kDa, a protein that is upregulated in the mitochondria of activated microglia. Binding of [^11^C]-(R)-PK11195 or similar tracers may, therefore, be a biomarker of microglial activation as it is expected to be higher in areas of the brain with significant inflammation or neuronal damage (70).

Using [^11^C]-(R)-PK11195 PET imaging, Holmes and colleagues (71) published the first *in vivo* study examining microglial activation and suicidal thinking. The authors observed increased TSPO density in the individuals with MDD compared to HC subjects, with the most pronounced finding in the ACC. They also found that TSPO expression was significantly elevated in the ACC and insula in patients with suicidal thoughts compared to patients without suicidal thoughts. The authors concluded that the greater TSPO binding in people with MDD and suicidal thoughts supported what four of the eight post-mortem studies had observed regarding a positive relationship between microglial activation and SB. It was also postulated that there was a higher specificity of microglial activation for suicide over psychiatric diagnoses.

#### Limitations

The authors, however, were unable to distinguish between the resting and activated phenotypes of microglia and, therefore, could not infer any underlying mechanisms. Currently, TSPO PET imaging is considered the only valid modality for examining microglial activity *in vivo*; however, there are some additional drawbacks to this imaging technique that must be discussed. Though TSPO binding is thought to exclusively reflect microglial activation (72), some argue that under certain circumstances, it can be expressed when astrocytes are activated as well (73). Moreover, the radioligand, [^11^C]-(R)-PK11195, used in the above PET study is limited due to the short half-life of carbon-11, high nonspecific binding, high plasma protein binding, and low brain penetration (74). [^11^C]-(R)-PK11195 PET imaging, in addition, requires a brain region with no specific binding of TSPO as a reference region in order to quantify the *in vivo* binding. Nevertheless, no region is completely devoid of TSPO expression, as it is thought to be present throughout the entire brain (70, 75).

Due to these problems, second generation TSPO radiotracers have been synthesized to offer a superior quality for quantifying *in vivo* TSPO expression. These second-generation radioligands include [^18^F]-FEPPA (76), [^18^F]-PBR06 (77), [^18^F]-FEDAA1106 (78), [^11^C]-DAA1106 (79), [^11^C]-DPA-713 (80), [^18^F]-PBR111 (81), [^18^F]-DPA-714 (82), and [^11^C]-PBR28 (83). [^18^F]-FEPPA is promising, for example, given that it displays an appropriate metabolic profile, a high affinity for TSPO, good pharmacokinetics, and high brain penetration (74). However, given the sensitivity of second-generation tracers to a single nucleotide polymorphism in the TSPO gene, third-generation TSPO radiotracers are also being developed that would be insensitive to this polymorphism. Examples of third-generation radioligands are [^18^F]-GE180 and [^11^C]-ER176; nonetheless, the safety and efficacy of these compounds in humans have not yet been established (84, 85).

In sum, TSPO PET imaging, specifically [^11^C]-(R)-PK11195 PET, has several limitations that may affect the findings of studies using this modality, including the aforementioned study. More recent TSPO second-generation radiotracers are recommended for future studies investigating microglial activity *in vivo*.

### Mechanisms Connecting Psychosocial Stress, Microglia, and SB in Human Studies

The most recent neurobiologically inclined stress-diathesis model proposed that life stressors or trauma lead to neurobiological alternations, such as deceased serotonergic neurotransmission, dysregulated HPA axis responsiveness, and reduced CSF oxytocin levels that may then result in behavioral alterations (18). This reconceptualized model is undoubtedly helpful in shedding additional light on the mechanisms by which early stressors contribute to the vulnerability to SB in later adolescence and adulthood. The model does not, however, include the growing evidence regarding the association between microglial dysregulation and SB. To address this, we evaluated eight immunohistochemistry studies and one TSPO PET imaging study that directly discuss microglial over-activation in relation to SB. Now, for this section, we further explore the neurobiological mechanisms that were proposed by some of these studies and also provide an updated stress-diathesis model of SB.

In 1969, a study introduced the “serotonin hypothesis”, a theory which proposes that dysregulation in the metabolic pathway of the essential amino acid, tryptophan (TRY), is a significant risk factor for depression. Specifically, this theory suggests that there is a dysregulated shift, using TRY as a precursor, from serotonin synthesis to KYN synthesis (86). The dysregulated shift is theorized to occur due to the up-regulation of indoleamine 2,3-dioxygenase (IDO1). This enzyme becomes activated due to increased hormones and/or proinflammatory cytokines, including IL-1β, IL-6, TNF-α, and interferon gamma (IFN-γ) (87–89). By consistently stimulating the shift from serotonin synthesis to increased KYN synthesis *via* the KYN pathway, systematic IDO1 activation ultimately leads to depressive-like behavior (87). Nevertheless, the exact mechanisms that underlie how this dysregulation leads to depressive-like outcomes and/or SB remain unclear.

Steiner and collaborators (62) primarily tackle this conundrum by proposing that increased KYN contributes to serotonin depletion and a higher production of the excitotoxic metabolite QUIN, both of which result in behavioral changes. Both this team and others (63) suggest that depleted serotonin levels may be caused by activated microglia that have enhanced metabolism of TRY to QUIN through the KYN pathway (62, 63). Given that previous literature has found low serotonergic functioning to be associated with increased impulsivity and aggressive behaviors in adults (6), the finding that activated microglia decrease serotonin levels provides some understanding as to how microglial activation influences depressive-like behavior pertinent to SB. Moreover, a recent study has shown that reduced serotonin level is associated with an increased risk for SB despite adjustments for depression severity, further suggesting that serotonin depletion *via* KYN dysregulation may impact suicidality directly, regardless of a depression diagnosis (90).

Beyond serotonin reduction, the shift from TRY to QUIN is also posited to detrimentally affect the metabolism of kynurenic acid (KYNA), an N-methyl-D-aspartic acid (NMDA) receptor antagonist that is considered neuroprotective due to its inhibitory effect on glutamate neurotransmission (91, 92). In fact, reduced concentrations of KYNA have been correlated with excitotoxicity by QUIN (93). QUIN is considered excitotoxic because it is an agonist of the NMDA receptor; therefore, an increase in QUIN can lead to excessive NMDA signaling or glutamatergic dysregulation (94, 95). These mechanisms, as postulated by Schnieder and colleagues (63), can potentially disrupt the BBB. As a result, QUIN can heighten the stimulation of glutamatergic neurons, particularly in the ventral prefrontal cortex, causing a decrease in normal neuron connectivity within the limbic system and, subsequently, reduced impulse control. In addition to being a direct NMDA receptor agonist, QUIN has pro-oxidant properties that worsen the neurotoxic effects by corticosterone and pro-inflammatory cytokines, as well as pro-inflammatory capabilities due to enhancement of the IFN-γ/IL-10 ratio (68). The adverse consequences of elevated QUIN are theorized to result in depressive features and/or SB. While astrocytes appear to predominantly catabolize KYNA from KYN, microglia instead transform KYN into 3-hydroxy kynurenine and QUIN (96, 97). This suggests that the above-mentioned mechanisms by which QUIN negatively affect the CNS may primarily be attributed to activated microglia.

Furthermore, an increase in IL-6, which is produced by microglia, is predicted by Schiavone and investigators (64) to enhance expression of NOX2 and NOX2-derived ROS production in GABAergic neurons. This subsequently causes altered GABAergic neurotransmission and thereby a loss of inhibition of glutamatergic neurons. The loss of inhibitory tone on the glutamatergic neurons contributes to excitotoxicity from increased glutamate release. This mechanism is hypothesized to also result in behavioral manifestations corresponding with SB; however, the authors do not specify which behavioral alterations may be affected.

### Potential Role of Microglial Cells in Reconceptualized Model of SB

**Figure 2:**
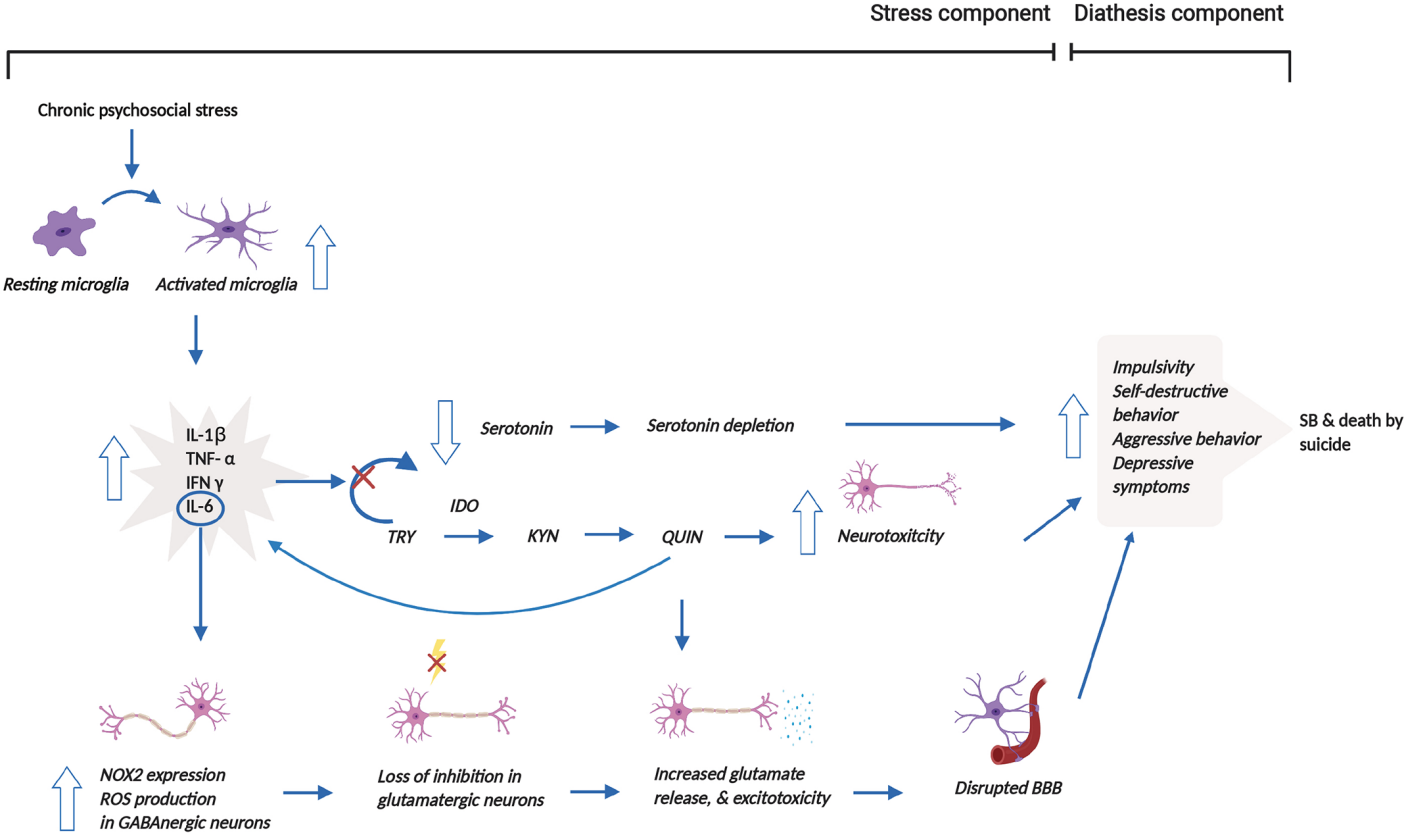
Reconceptualized clinical model of SB incorporating microglial dysregulation. This model proposes that prolonged psychosocial stress leads to the over-activation of microglia and associated release of pro-inflammatory cytokines. The release of pro-inflammatory cytokines stimulates the shift from serotonin to KYN synthesis through the induction of IDO1. This shift results in the depletion of serotonin. The production of QUIN from KYN synthesis, in addition, increases neurotoxicity. Moreover, increased QUIN production directly leads to heightened glutamate release and associated excitotoxicity but also indirectly causes this elevation by increasing IL-6 levels. Increased glutamate release and excitotoxicity contribute to the disruption of the BBB. Altogether, all these mechanisms contribute to behavioral manifestations, which may then result in SB and/or death by suicide. This figure was created with BioRender.com. BBB, blood brain barrier; IDO1, indoleamine 2,3-dioxygenase; IFN-γ, interferon gamma; IL-1β, interleukin-1β; IL-6, interleukin-6; NOX2, NADPH oxidase; KYN, kynurenine; QUIN, quinolinic acid; ROS, reactive oxygen species; SB, suicidal behavior; TNF-α, tumor necrosis factor-α; TRY, tryptophan.

Based on the aforementioned mechanisms, we propose that psychosocial stress, one of the stress components of the stress-diathesis theory, induces microglial activation, which increases the release of pro-inflammatory cytokines, shifting from serotonin to KYN synthesis through the induction of IDO1 (62). Subsequently, the catalyzation of TRY by IDO1 produces QUIN, which disrupts glutamatergic neurotransmission, disrupting the BBB and ultimately leading to reduced impulse control through reduced neuronal connectivity within the limbic system (63). Production of QUIN by activated microglia has pro-inflammatory and pro-oxidant properties that additionally contribute to increased neurotoxicity (68). Microglial activation also, in part, leads to enhanced IL-6 release, which causes elevated NOX2 expression and NOX2-derived ROS production, resulting in a loss of inhibition in glutamatergic neurons. These series of reactions affect glutamatergic neurotransmission by increasing glutamate release. The increased excitotoxicity due to the higher glutamate release then contribute to the altered behavior that is associated with SB (64). Thus, the changes in serotonergic and glutamatergic neurotransmission mentioned above lead to behavioral changes. These altered behavioral traits in suicide victims do not appear to be diagnosis specific (64, 68), thereby constituting a diagnosis-independent diathesis for SB. **Figure 2** illuminates these mechanisms and the interactions between the stress and diathesis components of the clinical model of SB.

### Blood and Cerebrospinal Fluid Markers, Depressive Outcomes, and SB

A number of human studies have also investigated the relationship between metabolites of the KYN pathway and depressive features and/or SB by measuring these metabolites in the blood or CSF. Eleven studies of the KYN pathway metabolite, KYN, provided no convincing evidence of differences in blood KYN levels between depressed patients without SB relative to healthy controls (91, 98–107). On the other hand, seven studies reported lower blood KYN concentrations in the depressed group (108–114). A meta-analysis, which evaluated these studies, similarly concluded that blood KYN levels were lower in depressed patients compared to HC (115). Moreover, the study by the Setoyama group found that blood KYN levels were negatively correlated with suicidal ideation in depressed patients (114). By contrast, Sublette and colleagues demonstrated that blood KYN concentrations were elevated in suicide attempters compared to non-suicidal patients (107). Another study reported a higher blood KYN/TRP ratio in MDD subjects with SB versus MDD subjects without SB or HC. Results indicated that the KYN/TRP ratio was positively correlated with the severity of suicidality in patients with histories of SB (116). Brundin et al. (117) additionally identified a higher ratio of CSF KYN/TRP in patients with prior suicide attempts relative to HC. Altogether, these results suggest that higher concentrations of KYN have a greater association with SB compared to the relationship with depression.

Twelve studies examining KYNA, another metabolite of the KYN pathway, found no significant differences in blood KYNA concentrations between patients with depression without SB and HC (91, 99, 100–102, 105, 106, 108–110, 118, 119). One study, in addition, reported no differences in levels of CSF KYNA among depressed patients and HC (120). Four studies, however, provided evidence of reduced levels of KYNA concentrations in depressed patients relative to HC, with three studies reporting the result based on KYNA levels in blood (98, 111, 121) and one from KYNA levels in the CSF (120). Also, a meta-analysis that reviewed the above-mentioned studies measuring blood and CSF KYNA likewise concluded that concentrations of this metabolite were reduced in patients with depression in comparison to HC (115). On the other hand, one study reported that psychiatric patients who made previous suicide attempts rather than simply endorsing an MDD diagnosis had reduced levels of KYNA in the CSF when compared to HC (122). These studies evaluating KYNA showed largely no differences between study and control groups for either depressive-like behaviors or SB.

Several studies have also measured levels of the KYN pathway metabolite, QUIN, in the blood or CSF in patients with depression. Among these investigations, six studies identified no group differences in blood QUIN concentrations between depressed patients and HC (99, 100, 102, 105, 106, 115), while three found elevated levels of QUIN in patients with depression, with two reporting the finding after measuring QUIN levels in blood (91, 98) and another from measuring QUIN levels in CSF (120). A meta-analysis, which also reviewed the above studies, additionally revealed that blood QUIN levels were higher in the depressed group, although the effect size was small (115). Moreover, Bay-Richter and colleagues reported greater concentrations of CSF QUIN in patients with prior histories of suicide attempts compared to HC (122). A more recent study found a decreased ratio of CSF picolinic acid (PIC), an additional neuroprotective metabolite of the KYN pathway, to QUIN in subjects who attempted suicide relative to HC (117). These results indicate that a lower PIC/QUIN ratio could potentially represent a marker of suicidality; however, these results remain preliminary and await further research. Although the majority of investigations examining QUIN relevant to depression found no significant results, the studies that analyzed QUIN in relation to SB conversely reported findings that were significant when compared with HC.

To summarize, there have been many studies of blood or CSF markers of the KYN pathway that may relate to depression and/or SB. Of these studies, a number of them investigated metabolites of the KYN pathway (e.g., KYN, KYNA, QUIN) in relation to depression; however, most studies did not find significant results. Among studies that sampled patients with SB, results predominantly showed significant results, such as elevated levels of blood or CSF KYN/TRP ratio and CSF QUIN, indicating perhaps that the upregulation of the KYN pathway may be associated with suicidality. However, the number of studies evaluating this relationship was limited and more research is needed.

## Conclusions

In the past decades, several theories have been proposed to conceptualize the etiology of SB; particularly, in recent years, many studies have focused on investigating the neurobiological aspects of this complex, multifactorial phenomenon. Through this review, we examined the original immunohistochemistry and neuroimaging studies that analyzed the relationship between microglial dysregulation and SB. Based on their results and proposed mechanisms, we were then able to construct a reconceptualized stress-diathesis theory of SB that incorporates the role of microglial activity. In our updated model, we suggest that chronic microglial activation may play a mediating role in the etiology of SB. That is, microglial dysregulation may be triggered by environmental challenges, namely psychosocial stressors, and then mediate several pathological neurobiological pathways that lead to behavioral changes and ultimately the outcome of SB.

The neurobiological pathways illustrated in this model predominantly involve dysfunction in the KYN pathway. As previously discussed, the results from studies, which evaluated the concentrations and/or ratios of various metabolites of the KYN pathway, also showed that SB may be associated with KYN dysregulation. Such findings are consistent with the results and hypothesized mechanisms from studies of microglial over-activation in suicidality that were examined in this review; nevertheless, it should be noted that the number of studies measuring peripheral blood and/or CSF metabolite levels of the KYN pathway in patients with SB are highly limited at this time. Research that further investigates these metabolites in suicidal patients is, therefore, necessary. It would also behoove investigators to test the efficacy of currently available pharmacotherapeutics in normalizing KYN dysregulation in suicidal individuals.

We synthesized our updated clinical model of SB by evaluating the specific hypothesized neurobiological pathways discussed in the studies that were included in this review. However, advances in the fields of neurobiology and epigenetics have revealed that other mediating factors, namely HPA axis responsiveness, blunted cortisol, and oxytocin are also related to the outcome of SB (9, 11–14, 16, 17). These factors were not incorporated in our reconceptualized model but may be related to microglial dysregulation as well, thereby representing another area for which to conduct original research.

Also, it remains unclear whether microglial cells become primed due to early adverse experiences, thus making them more susceptible to prolonged activation in response to subsequent stressors later in life. In this case, we may speculate that chronic microglial activation would not only assume a role in instigating the diathesis for SB, which we outlined in **Figure 1**, but might provoke, later in time, the outcome of SB. In this case, future research, ideally from prospective or longitudinal study designs, is needed to definitively establish whether microglial dysregulation exclusively impacts the risk for developing the diathesis traits leading to SB or whether it also perpetuates SB at a later time in life.

All studies that were included in this review consisted of subjects who were either victims of suicide or participants who had suicidal thinking. None of the papers discussed the over-activation of microglia in relation to self-harming behavior, despite our inclusion of the keywords, “parasuicidal behavior”, “self-harming behavior”, and “self-harm”. Considering this paucity of information, it would be interesting to determine, likely through neuroimaging research, whether there is also a positive relationship between microglial activity and self-harm, as well as to inform through an updated stress-diathesis model how self-harm subsequently relates to the risk for death by suicide.

In this review, we observed an overarching number of immunohistochemistry studies in comparison to the singular neuroimaging study. It appears that PET studies that can detect microglial activity, such as the aforementioned TSPO PET imaging, are in their infancy and require further research and refinements. The field of suicidology would benefit greatly by enhanced radioligands that have improved signal-to-noise ratio and lower nonspecific binding. Second- and third-generation radiotracers show promise for future research investigations as they do display these favorable properties. Accordingly, using such radiotracers can allow for more definitive conclusions to be made from studies that evaluate microglial cells through TSPO PET imaging. It would also enable scientists to perform clinical trials that test anti-inflammatory psychopharmacological medications, such as minocycline, on humans with SB *in vivo*. This work would be valuable for elucidating the role of neuroinflammation in SB as well as prospectively providing an accessible treatment option to individuals who are at high risk of death by suicide. To reiterate, however, as SB is a highly complex phenomenon, extensive future research is warranted to further refine our understanding of the neurobiology of these behaviors before a targeted psychopharmacological intervention may be established.

## Author Contributions

NJK conceived and designed the study, reviewed the full-text of three articles that were deemed questionable for eligibility by PB, and made revisions to the manuscript drafts. PB reviewed the titles and abstracts of studies following database searches as well as the full-text of select articles, drafted the manuscript, and made edits to the manuscript drafts. All authors contributed to the article and approved the submitted version.

## Funding

This review article was funded by a Canadian Institutes of Health Research Clinician Scientist Salary Award and the Centre for Addiction and Mental Health Alternative Funding Plan, both awarded to NJK.

## Conflict of Interest

The authors declare that the research was conducted in the absence of any commercial or financial relationships that could be construed as a potential conflict of interest.

## Abbreviations

ACC, Anterior Cingulate Cortex; AD, affective disorder; AS, Asphyctic Suicide; BD, Bipolar Disorder or Bipolar Depression; BBB, Blood Brain Barrier; CSF, Cerebrospinal Fluid; CD45, Cluster of Differentiation 45; CD68, Cluster of Differentiation 68; CNS, Central Nervous System; dACC, Dorsal Anterior Cingulate Cortex; DBCBB, Douglas-Bell Canada Brain Bank; DCEM, Department of Clinical and Experimental Medicine; DLPFC, Dorsolateral Prefrontal Cortex; D-NOS, Depression Not Otherwise Specified; DPFWM, Dorsal Prefrontal White Matter; DRN, Dorsal Raphe Nucleus; HC, Healthy Control; HLA-DR, Human Leukocyte Antigen–DR isotype; HPA, Hypothalamic Pituitary Adrenal; Iba-IR, Ionized Calcium-binding Adaptor Molecule 1-immunoreactive; Iba-1, Ionized Calcium-binding Adaptor Molecule 1; IDO1, Indoleamine 2,3-dioxygenase; IFN-γ, Interferon Gamma; IL-1β, Interleukin-1β; IL-6, Interleukin-6; IL-10, Interleukin-10; IL-18, Interleukin-18; KYN, Kynurenine; KYNA, Kynurenic Acid; M1, Pro-inflammatory Pathway; M2, Anti-inflammatory Pathway; MBB, Magdeburg Brain Bank; MDT, Mediodorsal Thalamus; MDD, Major Depressive Disorder; NMDA, N-methyl-D-aspartic Acid; NO, Nitric Oxide; NOX2/MAC387, NADPH Oxidase and Macrophage Marker Antibody; NYSPIBC, New York State Psychiatric Institute Brain Collection; pACC, Pregenual Anterior Cingulate Cortex; PET, Positron Emission Tomography; PIC, Picolinic Acid; QUIN, Quinolinic acid; ROS, Reactive Oxygen Species; SB, Suicidal Behavior; SCZ, Schizophrenia; TNF-α, Tumor Necrosis Factor-α; TRY, Tryptophan; TSPO, Translocator Protein; UD, Unipolar Disorder; UF, University of Foggia; VPFWM, Ventral Prefrontal White Matter.
